# Prefiltered Single-Carrier Frequency-Domain Equalization for Binary CPM over Shallow Water Acoustic Channel

**DOI:** 10.3390/s22103821

**Published:** 2022-05-18

**Authors:** Ruigang Han, Ning Jia, Zhongyuan Guo, Jianchun Huang, Dong Xiao, Shengming Guo

**Affiliations:** 1 Institute of Acoustics, Chinese Academy of Sciences, Beijing 100190, China; hanruigang@mail.ioa.ac.cn (R.H.); tonyflair@163.com (Z.G.); huangjianchun@mail.ioa.ac.cn (J.H.); xiaodong@mail.ioa.ac.cn (D.X.); guoshengming7024@aliyun.com (S.G.); 2 Key Laboratory of Underwater Acoustic Environment, Chinese Academy of Sciences, Beijing 100190, China; 3 University of Chinese Academy of Sciences, Beijing 100049, China

**Keywords:** UWA communication, binary continuous phase modulation, single-carrier frequency-domain equalization, prefilter

## Abstract

The continuous phase modulation (CPM) technique is an excellent solution for underwater acoustic (UWA) channels with limited bandwidth and high propagation attenuation. However, the severe intersymbol interference is a big problem for the algorithm applying in shallow water. To solve this problem, an algorithm for prefiltered single-carrier frequency-domain equalization (PF-SCFDE) is presented in this paper. The regular whitening filter is replaced by a prefilter in the proposed algorithm. The output information sequence of this prefilter contains the forward information. To improve the performance, the output of the equalizer, combined with the forward information, is used to make the maximum likelihood estimation. The simulation results with minimum-shift keying and Gaussian-filtered minimum-shift keying signals over shallow water acoustic channels with low root mean square delay spread demonstrate that PF-SCFDE outperformed the traditional single-carrier frequency-domain equalization (SCFDE) by approximately 1 dB under a bit error rate (BER) of 10^−4^. A shallow sea trial has demonstrated the effectiveness of PF-SCFDE; PF-SCFDE had a reduction in BER of 18.35% as compared to the traditional SCFDE.

## 1. Introduction

Underwater acoustic (UWA) communication [1], which has great advantage over the wired communication, is an important technique for ocean research. However, the UWA channel is a complex time–space–frequency variable channel [2]. UWA communication is very challenging due to its characteristic of frequency-selective fading and limited bandwidth [3]. The continuous phase modulation (CPM) [4,5] technology is characterized by a constant envelope, high spectral efficiency, and noise robustness; it has been widely applied in radio communication [6,7,8,9]. Consequently, the application of CPM to UWA communication may enhance bandwidth and power efficiencies, thereby increasing the performance. Nevertheless, the received signal of a UWA CPM has obvious intersymbol interference (ISI) due to the frequency-selective fading of the UWA channel, so an effective equalization algorithm is required to reduce the ISI.

The traditional CPM signal equalization technology is a maximum-likelihood sequence estimation using the Viterbi algorithm [10], which is the optimal detection algorithm. However, the implementation of this algorithm is very challenging due to the fact that the complexity increases exponentially with signal and channel length. Using delayed decision feedback sequence estimation [11], the equalizer can reduce the sequence estimation complexity with a small performance degrade, but it is still complicated for long time delay channels. Consequently, numerous researchers have separated channel equalization and CPM detection, and used frequency-domain equalization (FDE) to simplify the processing. Because CPM is a nonlinear modulation scheme, a linear decomposition is required before the discrete Fourier transform (DFT). Tan et al. [12] deduced single-carrier FDE (SCFDE) coefficients for CPM signals based on Laurent [13] and orthogonal decompositions, and proposed a simple differential detection scheme for CPM signals with the modulation index of 1/2. Furthermore, the algorithm’s reliability was verified by using both minimum-shift keying (MSK) and Gaussian-filtered MSK (GMSK). Based on the Laurent decomposition, Pancaldi et al. [14] deduced the linear equalization, decision feedback equalization, and turbo equalization techniques of CPM in the frequency domain (FD). Simulation results suggested that the traditional FDE algorithm could provide an acceptable bit error rate (BER) with a reduced amount of computation, whereas the turbo equalization improved performance while also requiring a higher amount of computation. Van Thillo et al. [15] approximated the CPM autocorrelation matrix as a diagonal block matrix, simplifying the algorithm complexity without compromising performance. An analysis of the influence of the sampling interval on FDE performance was conducted, and Williams et al. [16] concluded that the best results can be obtained when three sampling points per bit are obtained. Because the effective channel impulse response is required in FDE, channel estimation is performed and analyzed based on SCFDE [17,18,19]. Among them, Van Thillo et al. [17] proposed a method replacing the cyclic prefix (CP) in FDE with the training sequence of the known symbol, which could perform synchronization and channel estimation under the same performance conditions.

CPM has not been widely used in UWA communication. Weber discussed the feasibility of blind equalization for MSK UWA communication [20]. Vadde [21] and Liu [22] studied MSK and GMSK signals for UWA communication, respectively, and employed the virtual time-reversal mirror to suppress multipath interference. To further enhance the performance of the binary CPM UWA communications, in this study we investigate the FDE technology for a CPM signal based on Laurent decomposition. From the perspective of SCFDE, a prefilter is specified, and a joint detector is consequently designed. Compared with the traditional SCFDE, the prefiltered SCFDE (PF-SCFDE) uses a prefilter to transform the matched filter output sequence into a sequence that includes partially correlated noise and forward information. The output information sequence of the prefilter reduces the interference of the backward information from the matched filter. Subsequently, logarithmic likelihood estimation (LLR) is employed at the detection end to perform a weighted fusion of the forward information, which can improve the performance. To verify the PF-SCFDE performance, simulations were performed using MSK and GMSK signals under various channel conditions. We realized that, with similar complexity, PF-SCFDE can effectively improve the system performance over the channel with a low root mean square (RMS) delay extension. A sea trial comparison and analysis of PF-SCFDE and SCFDE using MSK UWA communication is presented in this paper, verifying that PF-SCFDE has superior performance in eliminating ISI over shallow UWA channels.

The remainder of this article is organized as follows. Section 2 describes the binary CPM signal model and the transceiver structure of the UWA communication system. Section 3 presents the algorithm and symbol detection method of PF-SCFDE. In Section 4, MSK and GMSK are used to simulate and analyze the performance of PF-SCFDE under different channel structures. In Section 5, the performance of PF-SCFDE is analyzed using an MSK UWA communication sea trial. Finally, we conclude this study in Section 6.

## 2. System Description

### 2.1. CPM Signal with Laurent Decomposition

An amplitude-normalized CPM signal can be expressed as follows:

(1) s(t)=exp(jϕ(t)), t>0

where *ϕ*(*t*) denotes the phase containing the transmitted information; it is given by

(2) $$\phi (t)=2\pi h\sum_{n=-\infty }^{N-1}x_{n}q(t-nT),\;nT\leq t\leq (n+1)T$$

where **x** represents the transmission information, *T* denotes the symbol period, *q*(*t*) denotes the phase pulse function, and *h* represents the modulation index.

Because CPM is nonlinear, it needs to be linearly decomposed before being subjected to DFT. A decomposed binary CPM signal expression may be simplified to a single pulse amplitude modulation component according to the Laurent decomposition, as described by Darsena D et al. [23].

(3) $$s(t)=\sum_{n}b_{n}c(t-nT)$$

where *c*(*t*) represents the partial impulse response, and the pseudo symbol $b_{n}=\exp(j\pi h\sum_{i=0}^{n}x_{i})$ can be expressed in a recursive form *b_n_* = *jb_n_*_−1_*x_n_*.

### 2.2. Transceiver Structure

**Figure 1:**
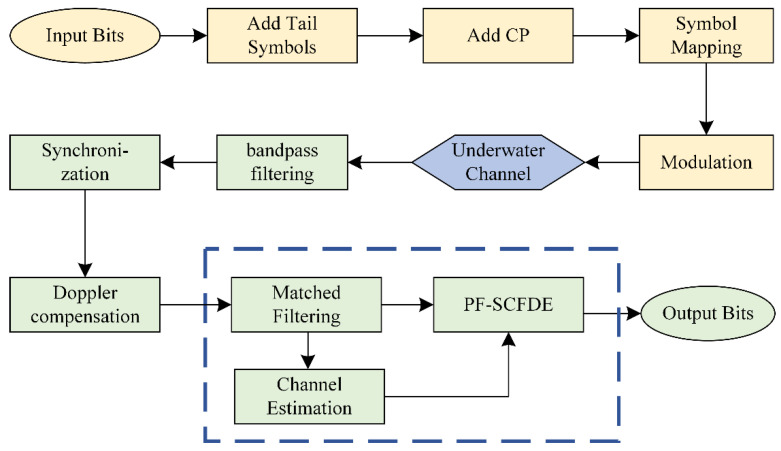
Block diagram of the CPM with the PF-SCFDE UWA communication system.

**Figure 2:**
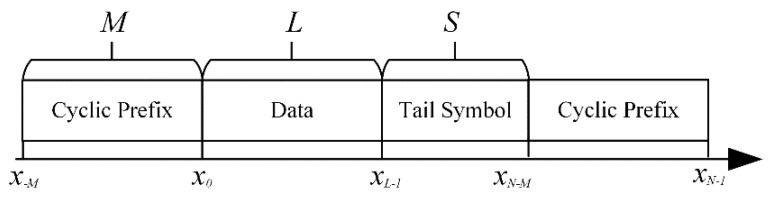
Complete frame structure.

The transceiver structure of the proposed CPM UWA communication system based on Laurent decomposition is shown in Figure 1. As shown in Figure 2, a frame structure of the transmitted MSK UWA communication signal is designed to construct the circulant matrix and estimate the channel. The transmission sequence **x** = {*x*_−*M*_, …, *x*_−1_, *x*_0_, …, *x_N_*_−2_, *x_N_*_−1_} comprises a CP, transmission information, and tail symbol, where *x_n_*∈ {±1}. The transmission information sequence has a length of *L*. A unique word sequence is employed to construct the CP **x**_uw_. As a recommendation, the length *M* of CP should be set to be longer than the maximum multipath delay of the channel, where the start and end states of **x**_uw_ should be equal to zero. With the addition of the tail symbol sequence of length *S*, the inclined phase path of **x** returns to zero at *n* = *N* − *M*, so that the phase continuity of the CPM signal is preserved, even after adding CP.

After the CPM signal has been transmitted through the UWA channel, it can be described as

(4) $$r(t)=\int_{-\infty }^{+\infty }h(\tau )s(t-\tau )d\tau +z(t)$$

where *h*(*t*) is the time-domain response of the UWA channel and *z*(*t*) is the zero-mean additive white Gaussian noise with variance *N*_0_.

At the receiver, the signal is passed through a bandpass filter first. Since we only consider the equalization algorithm, it is assumed that the received signal is synchronized and Doppler-compensated perfectly. Tan et al. [12] reported that the matched filter based on the Laurent decomposition of the CPM signal comprises a low-pass filter and a CPM coherent receiver based on *c*(*t*). Passing the received sequence **r** through the matching filter, the sequence can be expressed as

(5) $$\begin{array}{c} r_{n}=\int_{-\infty }^{+\infty }r(t)c(t-nT)dt\\ \;=\sum_{k}s(n-k)h_{k}+z_{n} \end{array}$$

(6) $$s_{n}≜\int_{-\infty }^{+\infty }s(t)c(t-nT)dt$$

(7) $$z_{n}≜\int_{-\infty }^{+\infty }z(t)c(t-nT)dt$$

With respect to the sparse nature of the UWA channel and the CP-based channel estimation method proposed by Van Thillo et al. [17], the front-end CP and the tail of **r** are extracted and combined before using the orthogonal matching pursuit (OMP) algorithm [24] to estimate the time-domain channel impulse response.

## 3. Prefiltered Frequency-Domain Equalization

**Figure 3:**
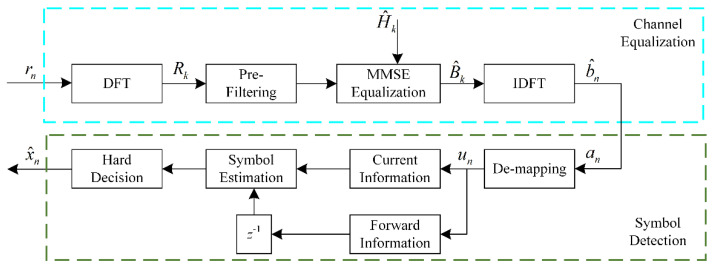
CPM receiver with PF-SCFDE.

The block diagram of the CPM receiver with PF-SCFDE is shown in Figure 3. As the matched filter output sequence r is converted to the FD, it is filtered by the equalizer. The noise whitening filter in the traditional SCFED is replaced by a prefilter in the proposed scheme. In that case, the output of the equalizer consists of the signal sequence, the partially correlated noise, and the forward information. Following the minimum mean square error (MMSE) equalization, the sequence is passed on to the symbol detector. As the modulation index is 1/2, the symbol detector de-maps the sequence to generate sequence **a**. By analyzing the memorized soft information *a_n_*, the real part represents the information about the current moment, whereas the imaginary part represents the symbol of the previous moment. Thus, weighted fusion and symbol estimation are performed with LLR based on the information contained in *a_n_*. Finally, the estimated symbols are dedifferentiated, and a hard decision is taken to get the transmission information. Additionally, for other modulation indices, the separation of forward information can be achieved by using the phase of ${\hat{b}}_{n}$.

### 3.1. Channel Equalization

The matched filter output sequence **r** in the FD can be obtained by taking the DFT of both sides of (5), as

(8) $$R_{k}=H_{k}S_{k}+Z_{k}$$

where *R_k_*, *H_k_*, *S_k_*, and *Z_k_* denote the FD representations of *r_n_*, *s_n_*, *h_n_*, and *z_n_*, respectively.

According to the CPM SCFDE algorithm, the noise term *z_n_* that is output by the matched filter correlates, so a noise whitening filter *w_n_* is required for whitening equalization. Despite that the traditional noise whitening filter *w_n_* can remove all relevant noise under optimal conditions, the filtered signal still possesses forward and backward information and performs symbol detection with the Viterbi algorithm or using only the current information.

In this study, an improved filtering framework is presented in which a new transfer function, *g_n_*, is used to define a prefilter, which reduces the energy of backward information while maintaining some correlated noise. As a result, sequence **r** can be converted into a signal sequence, **a**, with forward information, and then information at *n* and *n −* 1 can be used for symbol recognition simultaneously.

Applying the prefilter to the received signal *R_k_* yields

(9) $$R_{k}V_{k}=H_{k}S_{k}V_{k}+Z_{k}V_{k}$$

where $V_{k}=1/\sqrt{G_{k}}$, and *G_k_* is given by DFE of *g_n_*.

(10) $$g_{n}≜\left\{\begin{array}{l} \int_{0}^{+\infty }c(t+nT)c(t)dt\;\;\;,\;n=0\\ \sqrt{2}\times \int_{0}^{+\infty }c(t+nT)c(t)dt,\;n>0 \end{array}\right.$$

The equation can be expressed as follows:

(11) $${\hat{B}}_{k}=R_{k}V_{k}{\tilde{H}}_{k}$$

where an expression for the MMSE equalization coefficient ${\tilde{H}}_{k}$ is given by

(12) $${\tilde{H}}_{k}=\frac{{\hat{H}}_{k}^{*}V_{k}}{{\left|H_{k}V_{k}\right|}^{2}+N_{0}}$$

### 3.2. Symbol Detection

For the modulation index *h* = 1/2, the equalized signal ${\hat{b}}_{n}$ obtained by taking the inverse DFT on ${\hat{B}}_{k}$ can be de-mapped by

(13) $$a_{n}={\hat{b}}_{n}\exp(j\frac{\pi }{2}(n+1))$$

Because the real part Re(*a_n_*) and imaginary part Im(*a_n_*) of *a_n_* contain the information regarding the current and previous moments, respectively, Re(*a_n_*) and Im(*a_n_*) are extracted, respectively, and Im(*a_n_*) is delayed one symbol.

Denote *y_n_*∈{Re(*a_n_*), Im(*a_n_*)} and assume that Re(*a_n_*) and Im(*a_n_*) satisfy the Gaussian distribution after energy normalization. Then, *y_n_* can be expressed as follows:

(14) $$y_{n}=d_{n}+\sigma^{2}$$

where *d_n_*∈{±1} and *σ*^2^ denotes the variance of the output noise.

Hence, the conditional probability distribution function of *y_n_* can be expressed as follows:

(15) $$P(y_{n}=d_{n}|{\hat{x}}_{n})=\frac{1}{\sqrt{2\pi }\sigma }\exp(-\frac{{\left(y_{n}-d_{n}\right)}^{2}}{2\sigma^{2}})$$

Therefore, the log-likelihood estimate of the symbol *u_n_*, which is to be estimated, is given by

(16) L(un)=L(Re(an)=dn|x^n) + L(Im(an+1) = dn|x^n)=lnP(Re(an) = 1|x^n)P(Im(an+1) = 1|x^n)P(Re(an) = −1|x^n)P(Im(an+1) = −1|x^n)

The transmission information ${\hat{x}}_{n}$ can be obtained from a hard decision on *u_n_*.

(17) $${\hat{x}}_{n}=\left\{\begin{array}{l} 1,\;\;u_{n}u_{n-1}>0\\ 0,\;\;u_{n}u_{n-1}<0 \end{array}\right.$$

In the above calculation, *σ* can be calculated by the known CP information at the tail of the sequence **r**. The likelihood equation about *σ* is given by

(18) $$\frac{\partial }{\partial \sigma^{2}}\ln f(y_{n};b_{n},\sigma^{2})=-\frac{n}{2\sigma^{2}}-\frac{1}{2{\left(\sigma^{2}\right)}^{2}}\overset{n}{\underset{i=N-M}{\sum }}{\left(y_{i}-b_{i}\right)}^{2}=0$$

In addition, the maximum likelihood estimate of *σ*^2^ can be calculated by

(19) $$\sigma^{2}=\frac{1}{M}\sum_{i=N-M}^{N-1}{\left(y_{i}-b_{i}\right)}^{2}$$

For binary CPM signals whose modulation index is not 1/2, the symbol detection can be simplified [9]:

(20) $${\tilde{y}}_{n}=\frac{1}{\pi h}\arg({\hat{b}}_{n}{\hat{b}}_{n-1}^{*})$$

Based on the modulation index, the theoretical phase value of *x_n_* can be defined as ${\tilde{d}}_{n}=\pm \pi h$, and the conditional probability distribution function of ${\tilde{y}}_{n}$ is obtained:

(21) $$P(y_{n}={\tilde{d}}_{n}|{\hat{x}}_{n})=\frac{1}{\sqrt{2\pi }\sigma }\exp(-\frac{{\left(y_{n}-{\tilde{d}}_{n}\right)}^{2}}{2\sigma^{2}})$$

Moreover, the theoretical phase value of ${\hat{b}}_{n}$ without forward information can be given by $\arg({\tilde{b}}_{n})\in \{k\pi h\}$, where *k* is an integer. Considering that the forward information exists in ${\hat{b}}_{n}$ as phase, we can describe the phase interference caused by the forward information as follows:

(22) $$\gamma ≜\arctan(\frac{g(2)\sin(\pi h)}{1+g(2)\cos(\pi h)})$$

where *g* is the transfer function.

The conditional probability distribution function of forward information can be expressed as follows:

(23) $$P(\arg(b_{n+1})-\arg({\tilde{b}}_{n+1})=\gamma {\tilde{d}}_{n}|{\hat{x}}_{n})=\frac{1}{\sqrt{2\pi }\sigma_{b}}\exp(-\frac{{\left(\arg(b_{n+1})-\arg({\tilde{b}}_{n+1})-\gamma {\tilde{d}}_{n}\right)}^{2}}{2\sigma_{b}^{2}})$$

Then, the transmission information ${\hat{x}}_{n}$ can be obtained from a hard decision on the log-likelihood estimate L(*u_n_*) of the symbol.

(24) $$L(u_{n})\;=\;L(y_{n}={\tilde{d}}_{n}|{\hat{x}}_{n})+L(\arg(b_{n+1})-\arg({\tilde{b}}_{n+1})=\gamma {\tilde{d}}_{n}|{\hat{x}}_{n})$$

(25) $${\hat{x}}_{n}=\left\{\begin{array}{l} 1,\;\;u_{n}>0\\ 0,\;\;u_{n}<0 \end{array}\right.$$

### 3.3. Complexity Analysis

A comparison of the computational complexities of PF-SCFDE and SCFDE is presented in this subsection. *N* represents the length of the sequence after removing the front-end CP, and *M* represents the length of the tail-end CP. Because both algorithms use the same matched filter, only the complexities of the equalizer and detector are compared.

The computational complexities of the two equalization algorithms are listed in Table 1. PF-SCFDE replaces the whitening filter with a prefilter before the MMSE equalization, and hence the complexities of this step are approximately equal, whereas the complexities of the symbol detection slightly differ because the information from the imaginary domain is used in PF-SCFDE. Due to the fact that the most computationally intensive steps in both equalization algorithms are DFT, the complexity of PF-SCFDE is similar to that of SCFDE.

## 4. Numerical Simulation

In this section, MSK and GMSK are employed to verify the performance of PF-SCFDE, and the simulations are performed over the Rayleigh fading and typical channels, respectively. The performance is also compared with SCFDE. The data frame length of the simulated signal was 1024 bits, the CP length was 256 bits, the signal center frequency was 6 kHz, the bandwidth was 3 kHz, and the transmission rate was 2 kbps. The added noise was bandpass filtered additive white Gaussian noise. The channel estimation used the OMP algorithm in the simulation.

Both MSK and GMSK belong to the binary CPM, with a modulation index of 1/2. In addition, we used a GMSK signal with BT = 0.3 and a truncated pulse length *L* = 3. With Laurent decomposition, the transfer functions *g_n_* of MSK and GMSK can be expressed as follows:

(26) $$\begin{array}{l} g_{MSK}\;:\{1,\sqrt{2}/\pi ,0,\ldots ,0\}\\ g_{GMSK}:\{0.996,0.7255,0.0802,9.249\times 10^{-4},0,\ldots ,0\} \end{array}$$

### 4.1. Rayleigh Fading Channel

The simulation of a Rayleigh fading channel is used to examine the channel structure’s influence on the equalization performance. Assume that the channel comprises seven paths, the average delay is 0.5 ms, and the average power of each path decreases exponentially with increasing delay. In the simulation, we measure the performance under channels with different RMS multipath delay.

(27) $$\kappa =\sqrt{E[{\left(\tau -\bar{\tau }\right)}^{2}]}=\sqrt{\bar{\tau^{2}}-{\bar{\tau }}^{2}}$$

where the channel average delays $\bar{\tau }$ and $\bar{\tau^{2}}$ are given by

(28) $$\bar{\tau }=E[\tau ]=\sum_{i}\frac{p_{i}}{\sum_{k}p_{k}}\tau_{i}$$

(29) $$\bar{\tau^{2}}=E[\tau^{2}]=\sum_{i}\frac{p_{i}}{\sum_{k}p_{k}}\tau_{i}^{2}$$

with *p_i_* as the power of each channel path.

**Figure 4:**
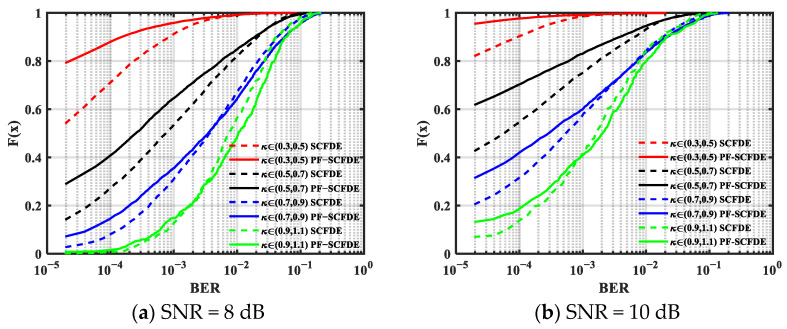
BER cumulative distribution function for MSK.

**Figure 5:**
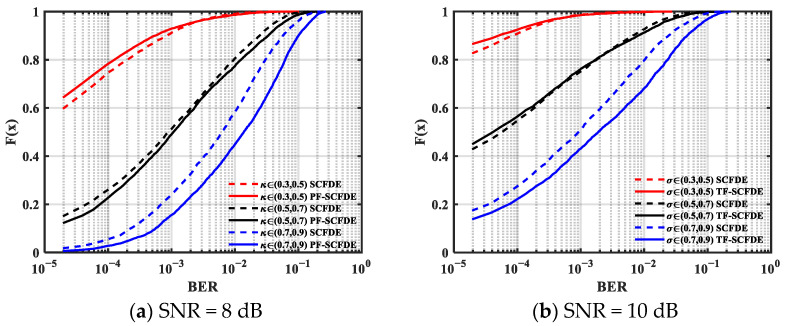
BER cumulative distribution function for GMSK.

A statistical analysis of the effect of *κ* on the performance of the two algorithms was performed. Figure 4 and Figure 5 illustrate the BER cumulative distribution function (CDF) of MSK and GMSK with a different SNR, respectively. PF-SCFDE is represented by the solid line, whereas SCFDE is represented by the dashed line. The performance of both equalization methods declines when *κ* increases.

From the simulation results, PF-SCFDE had better BER performance than SCFDE when *κ* was low. PF-SCFDE was superior to SCFDE for MSK transmission when *κ* < 0.9, and SCFDE had certain advantages when *κ* > 0.9. For GMSK, PF-SCFDE had better performance when *κ* < 0.5. Compared with MSK, owing to the longer pulse memory length (*L* = 3), the forward information contained other information except for time *n* − 1, so PF-SCFDE performance decreased.

### 4.2. Typical Channel

For the simulation, we chose the channel ChA provided by Tan et al. [12] and the channel ChB from UWA measured in the shallow waters of the South China Sea. Table 2 indicates the structure of ChA, and the delay is expressed as a symbol interval. It is shown that the maximum delay spread of ChA is 12.5 ms.

**Figure 6:**
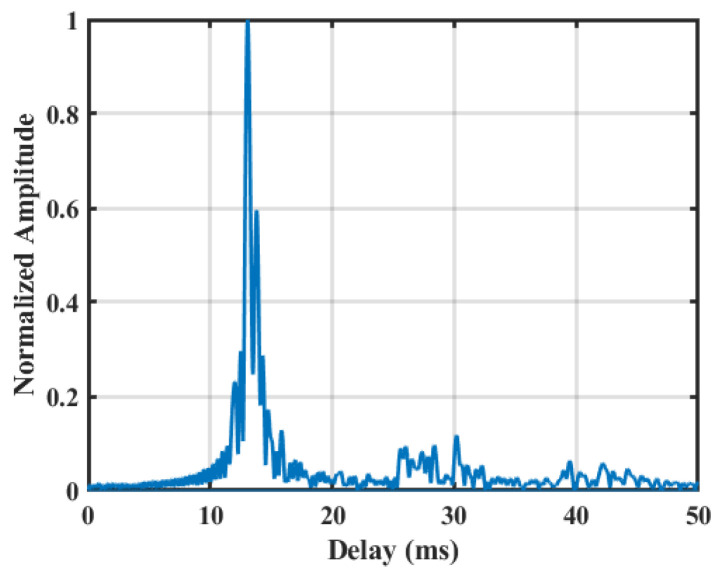
Measured channel (ChB) for simulation.

ChB was measured from a sea trial, in which the transmission range was approximately 5.3 km, the sea depth was 160 m, the transmitting depth was 80 m, and the receiving depth was 97 m. Figure 6 shows the time-domain response waveform of the measured channel with a delay spread of approximately 20 ms. It is evident that each path of the channel has a cluster structure [25] and that the channel can be divided into four main multipath clusters, among which there are multiple paths with larger energy near the main path. Since only the ability of the equalization algorithm to resist multipath interference is concerned, the time-varying and Doppler problems of general underwater acoustic channels are not considered in the simulation.

**Figure 7:**
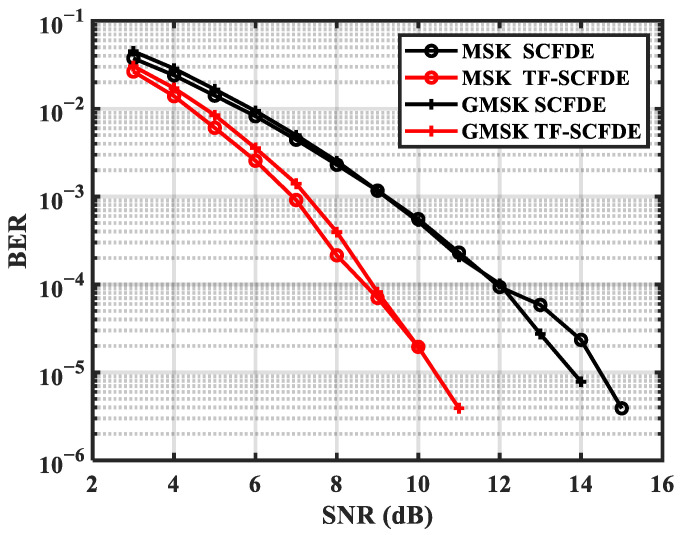
BER for ChA.

**Figure 8:**
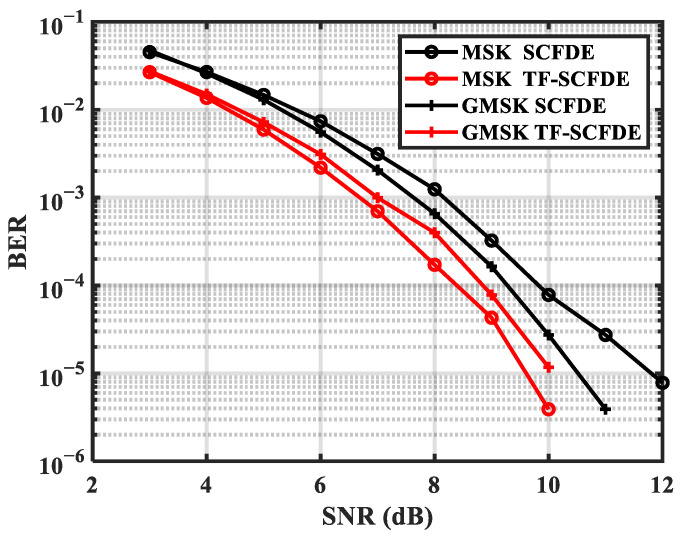
BER for ChB.

Figure 7 and Figure 8 show the performance of MSK and GMSK over two channels, respectively. For MSK and GMSK, PF-SCFDE performed approximately 3 dB better than SCFDE over ChA when the BER was 10^−4^. In addition, PF-SCFDE achieved 0.5 and 1.5 dB performance gains for MSK and GMSK, respectively, over ChB.

## 5. Sea Trial

**Figure 9:**
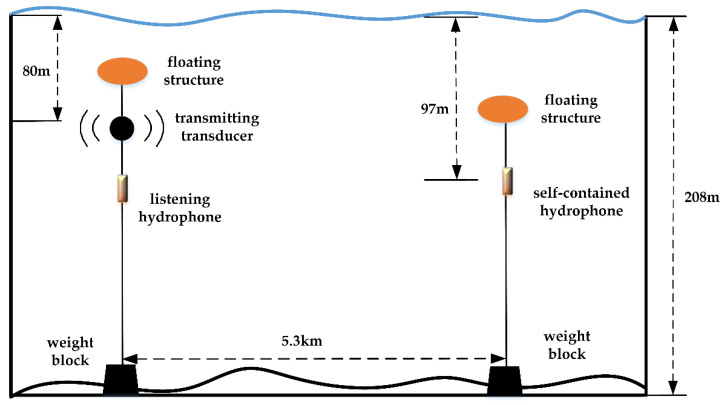
Sea trial program.

A sea trial was performed in the shallow waters area the South China Sea to evaluate the capabilities of PF-SCFDE. The sea depth of the test area was 208 m. As shown in Figure 9, the MSK UWA communication signal was transmitted using an acoustic transducer. The source level was divided into three grades, which are 196, 190, and 184 dB, respectively. The depth was approximately 80 m. The receiving signal was collected by a self-contained hydrophone located at a depth of 97 m, and the distance was approximately 5.3 km.

**Figure 10:**
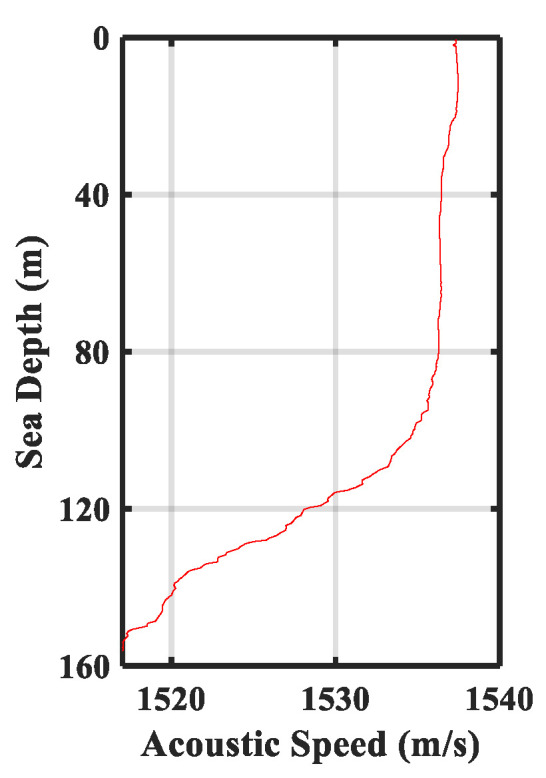
Measured sound velocity.

**Figure 11:**
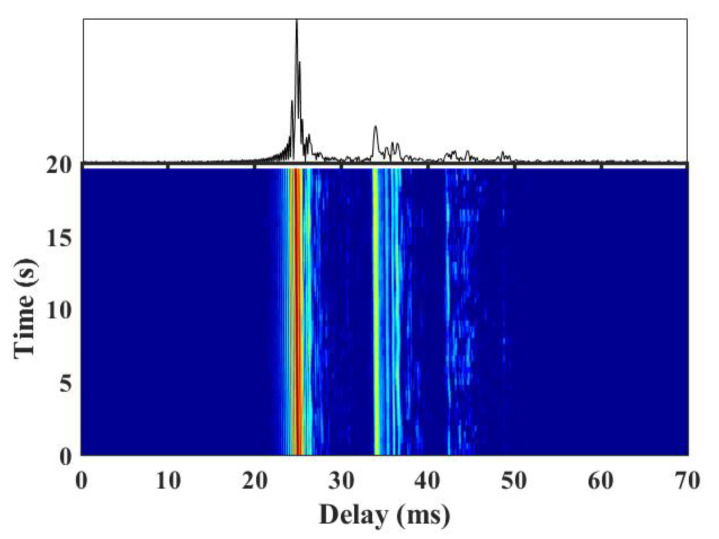
Measured channel in a certain period.

The sound velocity profile measured is shown in Figure 10. The velocity is close to constant in the upper layer of the water up to 100 m, and it has a negative gradient distribution in the lower layer. A total of 210 groups of signals were transmitted in the sea trial. In addition, a measurement of the channel is made by sending linear frequency modulation signals periodically in the experiment. Channels measured in a certain period are shown in Figure 11. The maximum channel delay was approximately 18 ms. The channels can be divided into four clusters. It is shown that the channel has a certain time variability, but the main path energy was relatively stable.

The center frequency of the transmitted MSK signal was 6 kHz, the transducer bandwidth was 4–8 kHz, the data communication rate was 2 kbps, and the transmission information sequence per frame was 256 bits. The received MSK signal was processed by SCFDE and PF-SCFDE, respectively.

Note that due to the signal transmitted in different times, the UWA channel changed during the sea trial, hence there was a significant difference in the received signal SNR. Table 3 shows the number of error bits in the differently received SNRs. It is shown that the number of error bits for PF-SCFDE and SCFDE reduces as the SNR increases, and the overall number of error bits for PF-SCFDE is smaller than that of SCFDE. In accordance with the different SNR intervals in the table, the error bits of PF-SCFDE are reduced by 33, 16, and 2. Based on the statistics of the 210 groups of received symbols of 5.376 × 10^4^ bits, the number of bit errors of SCFDE and PF-SCFDE was 278 and 227, respectively. PF-SCFDE outperformed SCFDE by 18.35%. The received signal is prone to phase shift due to the errors caused by the signal synchronization and Doppler frequency shift. In addition, PF-SCFDE is more sensitive to the phase shift due to the imaginary domain information is applied in the process.

## 6. Conclusions

An application of binary CPM is presented in this article to achieve the bandwidth and power efficiency improvements in the UWA channel. Based on SCFDE for CPM, and the output-memorized sequence of the equalizer, the PF-SCFDE for CPM is proposed. Compared with SCFDE, PF-SCFDE employs a prefilter to convert the information sequence output by the matched filter into an information sequence that contains forward information, thereby reducing the ISI caused by the backward information generated by the whitening filter. The LLR is used to estimate the forward information and make symbol detection, which can ultimately boost the receiver’s performance. The simulation results indicate that the PF-SCFDE performance is superior to that of SCFDE when the channel RMS delay spread is low. A sea trial demonstrated the ability of the PF-SCFDE to weaken multipath effects, which makes it a suitable communication scheme for shallow water acoustic channels.

## Figures and Tables

**Table 1:** Computational complexity comparison between SCFDE and PF-SCFDE.

Equalizer Type	Complex Multiplication	Complex Addition
SCFDE	2 *N* log_2_(*N*)+10 *N*	2 *N* log_2_(*N*)+*N*
PF-SCFDE	2 *N* log_2_(*N*)+11 *N+M*	2 *N* log_2_(*N*)+2 *N+M*

**Table 2:** Delay power profiles of ChA.

Delay (Symbol Interval)	0	1	2	8	12	25
**Power Fraction**	0.189	0.379	0.255	0.090	0.055	0.032

**Table 3:** Number of error bits of received signal frames in different received SNR.

SNR (dB)	Number of Frames	Error Bit of SCFDE	Error Bit of PF-SCFDE
5–10	22	212	179
10–15	52	58	42
15–20	72	8	6
>20	64	0	0
Total	210	278	227
